# Laparoscopic excision of proximal ileal GIST presenting with massive upper GI bleeding: a case report

**DOI:** 10.1097/RC9.0000000000000500

**Published:** 2026-04-29

**Authors:** Aashutosh Jha, Suman Adhikari, Sujan Shrestha, Sandesh Doranga, Sushil Mishra, Binod Bade Shrestha

**Affiliations:** Department of Surgery, Manipal College of Medical Sciences, Pokhara, Nepal

**Keywords:** case report, gastrointestinal bleeding, gastrointestinal stromal tumors, laparoscopy

## Abstract

**Introduction:**

Gastrointestinal stromal tumors (GISTs) are rare mesenchymal tumors of the gastrointestinal (GI) tract, most commonly arising in the stomach. Ileal GISTs are uncommon, and presentation with upper GI bleeding is exceptionally rare, posing a diagnostic challenge.

**Case presentation:**

We report a case of a 60-year-old woman presenting with hematemesis, melena, and severe anemia. After initial stabilization, upper GI endoscopy failed to identify the source of bleeding. Contrast-enhanced computed tomography revealed a heterogeneously enhancing mass in the proximal ileum. Laparoscopic-assisted resection of the ileal segment with end-to-end anastomosis was performed. Histopathology confirmed GIST, positive for c-kit and DOG1. The postoperative period was uneventful, and the patient remained symptom-free at 1-month follow-up.

**Discussion:**

Ileal GISTs presenting with acute upper GI bleeding are rare and often missed on routine endoscopy, highlighting the importance of imaging for diagnosis. Complete surgical excision with negative margins remains the standard treatment. Minimally invasive laparoscopic resection is safe, feasible, and effective. Adjuvant tyrosine kinase inhibitors, such as imatinib, are reserved for high-risk lesions or unresectable tumors.

**Conclusion:**

This case highlights the importance of considering GIST as a differential diagnosis in obscure upper GI bleeding. It also demonstrates that laparoscopic resection is a safe and effective treatment option for ileal GISTs, even in the context of acute bleeding.

## Introduction

Gastrointestinal stromal tumors (GISTs) are rare primary tumors of the gastrointestinal (GI) tract^[^1^]^ but represent the most common GI sarcomas. They are thought to originate from the interstitial cells of Cajal or their precursors^[^2,3^]^. Their molecular pathogenesis commonly involves activating mutations in receptor tyrosine kinases, or platelet-derived growth factor receptor alpha, which aids in distinguishing them from other mesenchymal tumors^[^4^]^. Almost half occur in the stomach and about one-third in the small intestine, with fewer cases in the colon or esophagus^[^5^]^. Within the small intestine, ileal GISTs are less common than jejunal GISTs^[^6^]^.

GISTs are usually asymptomatic and detected incidentally. If symptomatic, they present with nonspecific GI symptoms such as abdominal pain, distension, early satiety, nausea, or vomiting, and rarely a palpable mass^[^7^]^. GI bleeding is the most frequent complication of larger GISTs^[^8^]^ due to pressure necrosis and mucosal ulceration, presenting as hematemesis, melena, hematochezia, or anemia^[^9^]^. Occasionally, they may present as an acute abdomen from intra-abdominal bleeding requiring emergency surgery^[^5,10^]^. Contrast-enhanced computed tomography (CECT) is the preferred imaging modality, although ultrasound, magnetic resonance imaging (MRI), and positron emission tomography (PET) may also aid diagnosis^[^9^]^.

HIGHLIGHTS

- Proximal ileal gastrointestinal stromal tumors (GISTs) are rare and may manifest with upper gastrointestinal bleeding.
- Routine endoscopies may miss ileal lesion; contrast-enhanced computed tomography scan aids in diagnosis.
- Complete surgical excision with negative margins remains the standard treatment.
- Laparoscopy-assisted resection is safe and effective alternative to open surgery for ileal GISTs.
- Risk stratification guides the need for adjuvant therapy and follow-up.

Surgical resection remains the primary treatment, as conventional chemotherapy and radiotherapy are ineffective^[^10^]^. Tyrosine kinase receptor inhibitors such as imatinib are used as both neoadjuvant and adjuvant according to risk stratification^[^9^]^. When feasible, laparoscopic resection is preferred due to its minimally invasive nature and comparable oncologic outcomes to open surgery^[^11^]^.

## Methods

This case has been reported in accordance with the SCARE guidelines^[^12^]^.

## Presentation of the case

**Figure 1.**
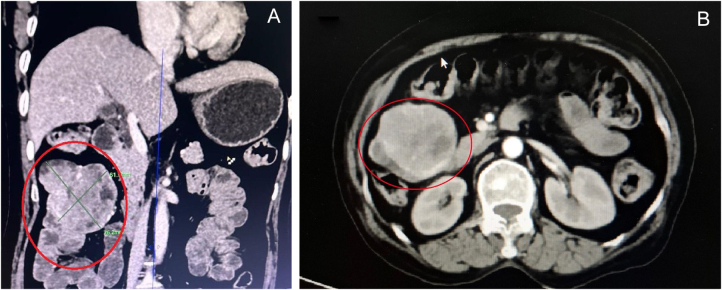
Contrast-enhanced computed tomography depicting a heterogeneously enhancing mass in upper right quadrant of abdomen (shown inside red ellipse). (A) Coronal section, (B) Axial section.

A 60-year-old non-alcoholic, non-smoker woman with no known comorbidities presented with a 1 month of intermittent abdominal pain, 2 days of melena mixed with fresh blood, and one episode of massive hematemesis (~1 cup) associated with light-headedness. She denied non-steroidal anti-inflammatory drugs or steroids use. On examination, she was pale and dehydrated but non-icteric. Blood pressure was 90/50 mmHg, pulse 112/min, respiratory rate 22/min, and oxygen saturation 93% on room air. Two wide bore intravenous lines were established, and rapid fluid resuscitation with normal saline was initiated, along with a proton-pump inhibitor infusion. Routine investigations revealed hemoglobin of 6.1 g/dl, and two units of packed red blood cells were transfused. Liver and renal function tests and coagulation profile were normal. Abdominal ultrasonography (USG) showed no abnormalities. Her Glasgow–Blatchford Score was 10, and she was admitted to the intensive care unit. Emergency upper GI endoscopy demonstrated antral gastritis without an identifiable bleeding source. Contrast-enhanced CT of the abdomen revealed a well-circumscribed heterogeneously enhancing mass measuring 6.2 × 5.7 × 5.2 cm in the right upper abdominal mesentery with small areas of necrosis (Fig. 1). Surrounding bowel loops were displaced, but other abdominal organs appeared normal, and no lymphadenopathy or ascites were present. The findings were suggestive of a GIST.

Surgical excision of the mass was planned after preoperative optimization. The patient was placed in the supine position under general anesthesia, and prophylactic antibiotics were administered prior to incision. Pneumoperitoneum was established via a 10-mm supraumbilical port, and a 5-mm working port was inserted in the left flank under direct vision to facilitate manipulation. Laparoscopic exploration of the abdominal cavity revealed a well-circumscribed exophytic mass arising from the proximal ileum and projecting toward mesentery. This likely accounted for the preoperative radiologic impression of a mesenteric mass. No peritoneal deposits or liver metastases were seen. The involved bowel loop was carefully mobilized using atraumatic laparoscopic bowel graspers, avoiding direct manipulation of the tumor surface. Special care was taken to prevent tumor rupture or capsular breach, with the mass handled indirectly through adjacent bowel to minimize the risk of tumor spillage.

Due to the size of the mass, the supraumbilical port incision was slightly extended along the midline to allow safe extracorporeal delivery of the affected bowel segment. The bowel segment containing the tumor was exteriorized through the extended incision. The mesenteric vessels supplying the involved segment were identified, ligated, and divided. Segmental resection of the small bowel encompassing the mass was then performed with 5-cm margins on either side. An end-to-end hand-sewn anastomosis was performed using 3-0 polydioxanone sutures. After ensuring hemostasis and bowel viability, the anastomosed segment was returned to the abdominal cavity. The peritoneal cavity was irrigated and inspected for hemostasis. A subhepatic drain was placed for postoperative monitoring of any collection. The abdominal wall was closed in layers, and sterile dressings were applied.

**Figure 2.**
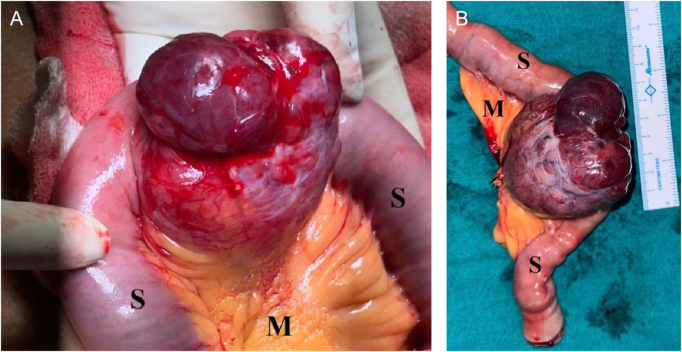
Gross image of a highly vascularized tumor arising from proximal ileum. (A) Intraoperative view during laparoscopic excision. The supraumbilical port was slightly extended to facilitate safe extraction of the intact tumor, without converting to an open procedure. (B) Resected specimen showing the tumor with attached small intestine (S) and mesentery (M).

Gross examination revealed a well-vascularized nodular mass measuring 7.0 × 6.0 × 3.6 cm with areas of hemorrhage and cystic degeneration (Fig. 2).

**Figure 3.**
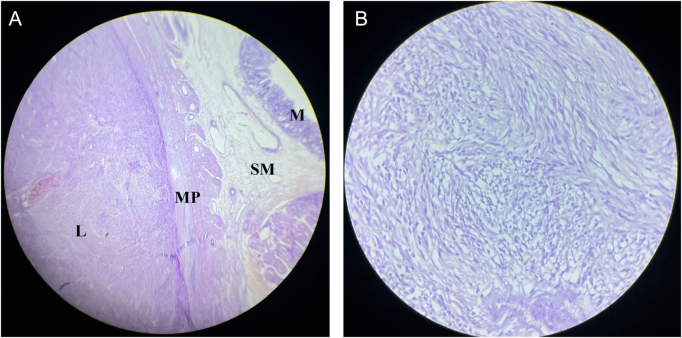
Histopathology showing (A) GIST lesion (L) arising from muscularis propria (MP) of ileum with intact mucosa (M), SM: submucosa. (B) High-power view: spindle cells arranged in interlacing bundles.

Histopathology showed a well-circumscribed spindle cell tumor arising from the muscularis propria, composed of interlacing fascicles of spindle shaped cells with eosinophilic cytoplasm and elongated nuclei. Mitotic activity was low [<5/50 high-power fields (HPF)], and no necrosis was identified (Fig. 3).

Immunohistochemistry demonstrated strong positivity for c-kit and DOG1, confirming the diagnosis of GIST. Spindle cell neoplasms of the small intestine have several differential diagnoses, including leiomyoma/leiomyosarcoma (SMA/desmin positive), schwannoma (S100 positive), and desmoid fibromatosis (β-catenin positive). In this case, the characteristic spindle cell morphology with dual positivity for c-KIT and DOG1 (markers highly sensitive and specific for GIST) was sufficient to establish the diagnosis^[^13^]^. Additional markers such as CD34, SMA, S100 and Ki-67 were not considered necessary. According to the American Joint Committee of Cancer prognostic staging, the tumor was classified as stage II, with a pathological TNM classification of pT3, i.e., locally advanced tumor; NX; Mx. The tumor was classified as high risk by the Modified National Institutes of Health (NIH) criteria and moderate risk by Armed Forces Institute of Pathology (AFIP) criteria as illustrated in Table 1.

**Table 1:** Risk stratification of the ileal GIST according to the Modified NIH and AFIP criteria with adjuvant therapy recommendation.

Parameter	Present case finding	Modified NIH interpretation	AFIP interpretation	Adjuvant imatinib rationale
Tumor site	Small bowel	Non-gastric	Small intestinal	Higher recurrence risk than gastric GIST
Tumor size	7 cm	5–10 cm group	5–10 cm group	Size ≥ 5 cm increases recurrence risk
Mitotic rate	<5/50HPF	Low (≤5)	Low (≤5)	Non-gastric GIST ≥ 5 cm
Tumor rupture	Absent	No rupture	Not included	Not contributory
Overall risk	-	High risk	Moderate risk	Adjuvant imatinib recommended

Based on the Modified NIH risk stratification criteria, non-gastric GIST measuring 5–10 cm with ≤5 mitoses/50 HPF is categorized as high risk. Current ESMO^[^14^]^ guidelines recommend adjuvant imatinib following complete resection.

Postoperatively, the patient was managed in the surgical intensive care unit. Oral sips were initiated on postoperative day (POD) 2, and advanced gradually with a soft diet on POD4. She was discharged on POD7 in stable condition. Adjuvant therapy with imatinib was initiated and planned for 3 years.

At 1 week outpatient follow-up, the surgical wounds were healing with no recurrence of GI bleeding. At 1 month telephone follow-up, the patient remained asymptomatic. Surveillance abdominal imaging was planned at 6 months after surgery.

## Discussion

GISTs are a diagnostically challenging cause of GI bleeding, particularly when clinical suspicion is low, as many arise beyond the reach of conventional endoscopy^[^15^]^. In our case, upper GI endoscopy failed to identify the bleeding source, and the lesion was detected on contrast-enhanced CT, highlighting the role of advanced imaging in obscure GI bleeding. Small bowel GISTs may require multiple diagnostic modalities such as videocapsule endoscopy, deep enteroscopy, or radiographic imaging (USG, CT, MRI, or PET-scan) for accurate localization^[^16,17^]^.

Although small bowel GISTs can cause GI bleeding, most reported cases involve the jejunum or present with lower GI bleeding. Massive upper GI hemorrhage from an ileal GIST is rare, as ileal lesions typically manifest with lower GI bleeding^[^18^]^. In our patient, acute massive upper GI bleeding with a negative endoscopy created a diagnostic challenge. Reports of successful laparoscopic resection in this acute setting remain limited, emphasizing both the rarity of this presentation and the feasibility of minimally invasive oncologic management. This case highlights the need to consider small bowel tumors in unexplained or recurrent GI bleeding and the value of a stepwise diagnostic strategy using advanced imaging when initial evaluations are inconclusive.

Table 2 summarizes selected previously reported cases of jejunal and ileal GISTs presenting with GI bleeding, compared with the present case.

**Table 2:** Selected reported cases of small bowel GISTs presenting with gastrointestinal bleeding.

Study	Age/Sex	Presentation	Diagnosis	Site	Treatment
Mahmoud & Salman (2020)^[^19^]^	54y/F	Melena, Hematochezia	CECT	Jejunum	Laparotomy and resection
Mohammed *et al*^[^20^]^	58y/F	Melena, upper abdominal pain	CT angiography	Jejunum	Laparoscopic resection
Liu *et al*^[^21^]^	32y/M	Hematemesis, abdominal pain	Diagnostic Laparoscopy	Jejunum	Laparotomy and excision
Ling *et al*^[^22^]^	89y/M	Melena, recurrent hematochezia	Capsule + CT	Ileum	Laparotomy and resection
Jha *et al*^[^18^]^	71y/M	Hematochezia, abdominal pain	CT angiography	Ileum	Laparoscopic excision
Khan *et al*^[^23^]^	59y/F	Syncope and melena	CT angiography	Ileum	Laparoscopic resection
Present case	60y/F	Hematemesis, melena, abdominal pain	CECT abdomen	Ileum	Laparoscopic excision

GISTs demonstrate variable biological behavior, with risk stratification based on tumor size, mitotic index, and site of origin. Prognostic systems such as NIH and AFIP incorporate these parameters to estimate malignant potential. In our case, the tumor measured 7 cm in the small intestine with low mitotic activity and no necrosis or atypia, corresponding to high risk by the Modified NIH criteria and moderate risk by AFIP criteria. Complete surgical excision with negative margins (R0 resection ≥1 mm) while minimizing tumor manipulation remains the standard treatment for localized GISTs^[^11^]^. This approach is supported by DeMatteo *et al*, who showed that tumor size and gross margin status, rather than microscopic margin status, primarily determine survival^[^24^]^. When feasible, laparoscopic resection is preferred, as it provides outcomes comparable or superior to open surgery^[^11^]^.

Organ sparing operation is preferred whenever possible. However, when complete resection would entail severe morbidity, e.g., total gastrectomy or hemicolectomy, neoadjuvant imatinib is recommended. Likewise, patients at high risk of recurrence are recommended for 3 years of adjuvant imatinib^[^25^]^. Neoadjuvant was deemed unnecessary in our case. However, adjuvant imatinib was initiated due to high-risk classification. The patient remained symptoms free during the short-term follow-up of 1 month, although this duration is insufficient to assess recurrence or long-term outcomes. Therefore, a structured surveillance protocol has been planned: CECT abdomen and pelvis every 6 months for the first 5 years, followed by annual imaging for the next 5 years, to facilitate early detection of recurrence^[^26^]^.

## Conclusion

This case highlights the need to consider GIST in obscure upper GI bleeding when endoscopy is inconclusive. It also demonstrates that laparoscopy-assisted resection with risk-guided adjuvant imatinib can successfully manage ileal GIST presenting with massive upper GI bleeding in the acute setting.
